# Total body irradiation is associated with increased burden of cerebral microbleeds in haematopoietic stem cell transplant recipients

**DOI:** 10.1111/bjh.70747

**Published:** 2026-08-12

**Authors:** Jure Hederih, Asad Charania, Camelia Vladescu, Ira Lacej, Richard Szydlo, Jiri Pavlu, Renuka Palanicawandar, Jose Ros‐Soto, Andrew Innes, Dragana Milojkovic, Eduardo Olavarria, Jane Apperley, Adam Waldman, Nichola Cooper

**Affiliations:** ^1^ Department of Haematology University College London Hospital, University College London Hospitals NHS Foundation Trust London UK; ^2^ Department of Haematology Hammersmith Hospital, Imperial College Healthcare NHS Trust London UK; ^3^ Department of Immunology and Inflammation Centre for Haematology, Imperial College London London UK; ^4^ Centre for Clinical Brain Sciences University of Edinburgh Edinburgh UK

**Keywords:** cerebral microbleeds, haematopoietic stem cell transplant, total body irradiation

Myeloablative conditioning (MAC) regimens for haematopoietic stem cell transplantation (HSCT) can include total body irradiation (TBI), which is associated with a wide range of acute and chronic toxicities.^1^ Cerebral microbleeds (CMBs) are haemosiderin deposits detected on magnetic resonance imaging (MRI) with detection heavily dependent on the field strength of the MRI machine, imaging protocols and experience of the interpreting radiologist.^2^ CMBs may develop after exposure to irradiation and have been reported at doses as low as 18 Gy in humans^3^ and 1.1–8.5 Gy, delivered through TBI, in non‐human primates.^4^ TBI doses used in MAC rarely exceed 15 Gy, but are delivered at the time of considerable physiological stress.^1^ Clinical relevance of CMBs remains controversial,^5^ but studies in patients receiving cranial irradiation for cerebral tumours have shown a correlation between CMBs and cognitive impairment^6^ and the presence of multiple CMBs has been linked to an increased risk of dementia,^7^ stroke and intracranial haemorrhage.^5^ While cognitive impairment is also a known late effect of allogeneic HSCT, its mechanisms remain largely unclear.^8^ There is presently no known association between TBI and CMBs in humans.

This observational study aims to assess the prevalence of CMBs in recipients of TBI‐based MAC compared to those receiving irradiation‐free MAC. Secondary analyses aim to explore the relationship between selected peritransplant stressors and CMB burden. The study has been approved by London‐Surrey Borders Health Research Authority Ethics Committee. All participants provided informed consent and we conducted the study in accordance with declaration of Helsinki. We recruited allogeneic HSCT survivors from the outpatient department of Hammersmith Hospital, London, England. Inclusion criteria were: previous MAC HSCT with either chemotherapy and TBI (TBI group) or chemotherapy alone (chemotherapy‐only group). The chemotherapy‐only group was recruited as a comparator, due to the fact that they have suffered from a primary pathology requiring an HSCT, received MAC and were at risk of similar post‐transplant complications. We excluded patients with known history of cranial irradiation, uncontrolled hypertension, insulin‐dependent diabetes or disease relapse. We excluded patients that could not communicate in English without an interpreter and those that were unable to consistently attend required follow‐up. The minimum required latency since HSCT was 3 years, as previous studies show that median latency from irradiation to developing CMBs is under 3 years.^9^ All patients meeting the inclusion criteria were considered for the study with 15 undergoing final data collection. Data on patient age, sex, diagnosis, body mass index (BMI), conditioning regimen, nadir platelet count, duration to engraftment (defined as three consecutive days of unsupported platelet count above 50 × 10^9^/L), graft‐versus‐host disease (GVHD), GVHD prophylaxis and haematopoietic cell transplantation comorbidity index (HCT‐CI)^10^ was collected from patient notes. All participants underwent a Susceptibility Weighted Imaging MRI scan with a 3‐T magnetic resonance machine (VB19; Siemens Verio, Erlangen, Germany) as per previously described dedicated protocol.^11^ The number of CMBs was reported by an experienced neuro‐radiologist blinded to the patient data. Mann–Whitney *U* test was used to assess the location shift in CMB numbers between groups, Fisher's exact test to compare prevalence of at least one or multiple (two or more) CMBs between groups and Spearman rank correlation in exploratory analysis of correlation between CMBs and selected peritransplant factors: age at transplant, latency since transplant, age at time of the scan, nadir platelet count, duration to platelet engraftment, HCT‐CI, BMI and acute GVHD grade. GraphPad Prism Version 11 was used for statistical analysis.

Fifteen patients were recruited: nine in TBI group and six in chemotherapy‐only group. Baseline characteristics are summarized in Table 1. These were largely similar; however TBI group had longer latencies since HSCT (median latency: 81 months, range: 40–149) compared to chemotherapy‐only group (median latency: 43.5 months, range: 40–72). Significantly longer latency since transplant in TBI group reflects the more recent introduction of irradiation‐free conditioning regimens in local practice. CMBs tend to persist once present^12^ and new lesions may occur after the initial insult,^13^ thus making this discrepancy in our baseline characteristics a potential confounder. However, our exploratory analysis did not show a significant correlation between latency since HSCT and number of CMBs (Figure 1B; Table S1). All TBI was delivered in fractionated, twice daily regimens. The median TBI dose was 13.2 Gy (range: 12–14.4 Gy) (Table S2). Supportive medication, including GVHD prophylaxis (Table S3) differed between the groups as a corollary of different conditioning regimens. All transplants occurred between December 2009 and February 2019. Example of a CMB is included in Figure S1.

**TABLE 1:** Summary of patient characteristics.

	Chemotherapy + TBI	Chemotherapy only
Total number of patients	9	6
Gender		
Female	6	3
Male	3	3
Conditioning		
CyTBI	8	0
Etoposide‐TBI	1	0
BuCy	0	2
TBF	0	4
Primary diagnosis		
AML	5	4
ALL	2	1
CML	2	1
Median age—years (range)	40 (32–61)	42 (27–62)
Median age at HSCT—years (range)	34 (22–49)	36.5 (23–58)
Median time from HSCT—months (range)	81 (40–149)	43.5 (40–72)
Median TBI dose—Gy (range)	13.2 (12–14.4)	0
Median time to platelet engraftment—days (range)	18 (13–73)	21.5 (16–44)
Median nadir platelet count—×10^9^/L (range)^a^	10 (5–41)	9 (4–18)
Platelet count at time of MRI scan—×10^9^ (range)^b^	242.5 (142–308)	200 (164–395)
GVHD (chronic or acute, any grade)	5	4
Median HCT‐CI	0 (0–1)	1.5 (0–3)
Median BMI kg/m^2^ (range)	22.3 (19.5–26.5)	22.8 (19.7–29.7)

Abbreviations: ALL, acute lymphoblastic leukaemia; AML, acute myeloid leukaemia; BMI, body mass index; BuCy, busulfan and cyclophosphamide; CML, chronic myeloid leukaemia; Cy, cyclophosphamide; GVHD, graft versus host disease; HCT‐CI, haematopoietic cell transplantation comorbidity index; HSC, haematopoietic stem cells; HSCT, haematopoietic stem cell transplant; MRI, magnetic resonance imaging; TBF, thiotepa, busulfan, fludarabine; TBI, total body irradiation.

^a^ One patient in TBI group was supported with platelet transfusions with a target of 50 × 10^9^/L due to need for ongoing anticoagulation in the context of multiple previous thrombotic events.

^b^ One patient in each group was unable to provide a blood sample at the time of the MRI scan and therefore had not had a platelet count determined.

**FIGURE 1:**
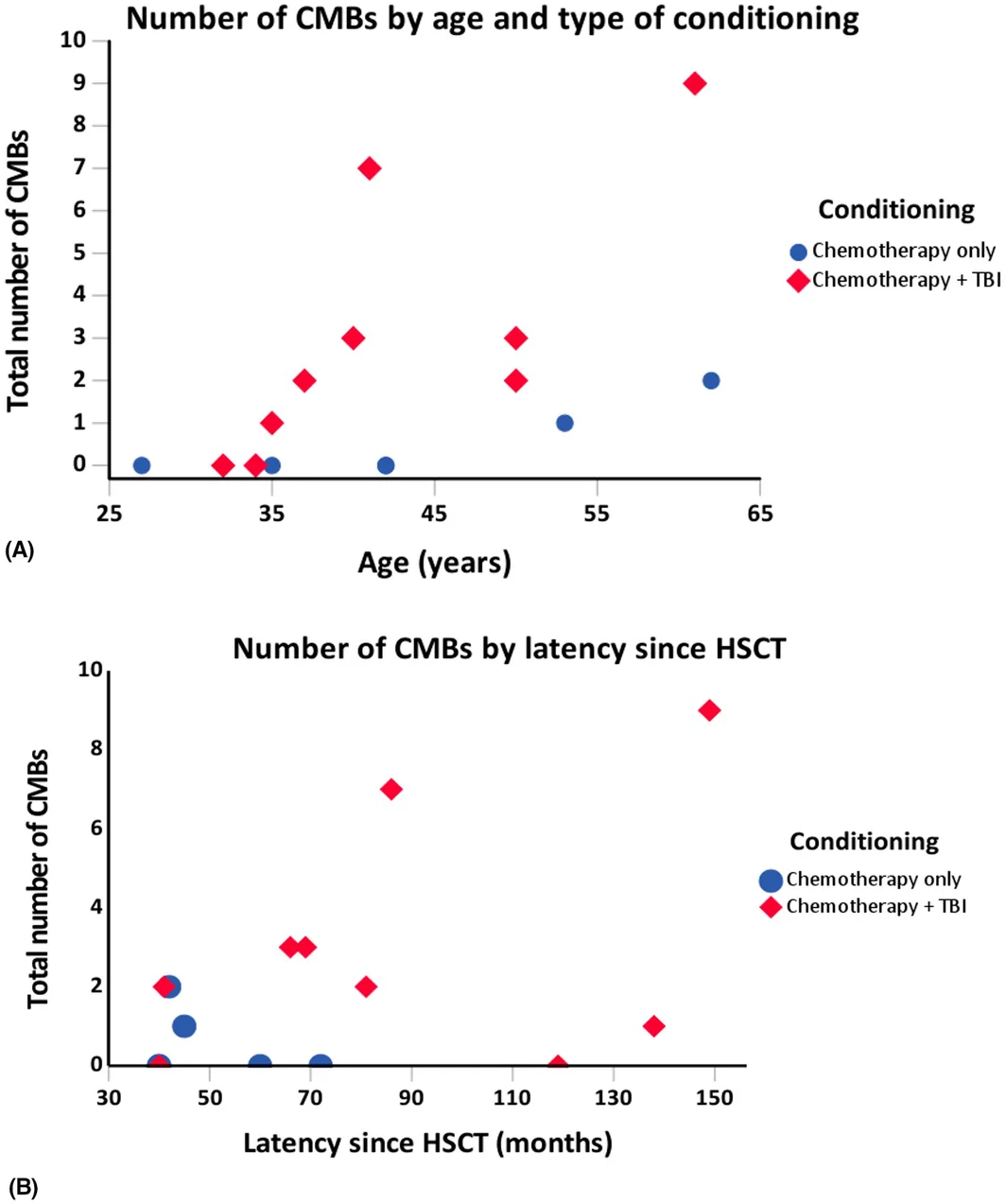
(A) Total number of CMBs and age in individual survivors, split by conditioning regimen. There is a higher propensity for younger TBI‐conditioned survivors to demonstrate multiple CMBs at a younger age. The prevalence of CMBs in HSCT survivors conditioned with chemotherapy‐only is arguably in keeping with numerous previous studies demonstrating low CMB prevalence in advanced age and absence in younger patients. Two patients in the chemotherapy‐only group were 42. (B) Total number of CMBs as related to latency since HSCT. There is no clear relation between increased latency since transplant and total number of CMBs. Two patients in chemotherapy‐only group had a latency of 40 months. CMB, cerebral microbleeds; HSCT, haematopoietic stem cell transplantation; TBI, total body irradiation.

CMBs were present in seven of the nine patients in TBI group (78%) and two of the six patients in chemotherapy‐only group (33%) (*p* = 0.14). Median number of CMBs was 2 in TBI group (range: 0–9) and 0 in chemotherapy‐only group (range: 0–2) (*p* = 0.05) (Figure 1A). Six (67%) patients in TBI group had multiple CMBs, compared to one in chemotherapy‐only group (17%) (*p* = 0.12) (Figure 1A,B). These data provide the first evidence of increased median CMB burden in HSCT survivors who have undergone conditioning with TBI. Higher overall prevalence and higher prevalence of multiple CMBs are also noted in TBI recipients for the first time, though this did not quite reach statistical significance. This is also the first study reporting an association between CMBs and irradiation doses below 18 Gy. It is possible that the peritransplant period conveys increased susceptibility to CMB formation thus allowing a lower dose of irradiation to trigger CMB formation. We hypothesize that we are observing a synergistic effect between the patient's underlying disease, a number of peritransplant stressors and TBI. We attempted to further characterize the association of selected peritransplant factors and the number of CMBs; however latency since HSCT (Figure 1B), severity and duration of thrombocytopenia, acute GVHD grade, HCT‐CI and age at transplant did not individually show a correlation with CMB burden (Table S1). Only one CMB in our study occurred in a patient with history of asparaginase or high‐dose methotrexate exposure. No patients had a history of thrombotic microangiopathy or microangiopathic haemolytic anaemia. One patient was receiving therapeutic anticoagulation. Low BMI has been shown to predict microangiopathy after TBI‐conditioned HSCT^14^; however, our study found no association between BMI and number of CMBs (Table S1). These findings should be treated as exploratory and further assessed in appropriately powered studies. CMB burden is associated with disease duration and nadir platelet count in immune thrombocytopenia,^11^ which is in contrast to our findings, but may be attributed to different pathophysiology of CMB formation.

In general population, increased CMB prevalence is associated with advanced age^12^; however, we observed a propensity for CMBs to develop at an earlier age in patients conditioned with TBI.

The 2 patients in chemotherapy‐only group that developed CMBs were 53 (single CMB) and 62 (2 CMBs) years old (median age: 57.5 years). Six patients in TBI group developed multiple CMBs (median age: 45.5 years, range: 37–61 years), with five of those being 50 years old or younger (Figure 1A). Previous studies in community‐dwellers show that prevalence of a single CMB at 50–55 years of age is 11.5% and that of multiple CMBs at 60–69 years of age is 4.9%.^12^ We also noted a statistically significant correlation between age at time of scan (but not at time of transplant) and CMB number (Table S1). The findings in the chemotherapy group may therefore, at least partially, be attributed to age rather than history of TBI. On the other hand, the prevalence of multiple CMBs in relatively young patients in TBI group is unusual. Multiple CMBs in younger individuals are extremely rare in community‐dwellers—a previous study using identical imaging parameters showed no CMBs in healthy controls with a mean age of 45.4 years (range: 21–64).^11^ The prevalence of CMBs in another study was 0.7% between ages of 45 and 50; however the scanner used lower field strength and utilized different imaging sequences, which could affect sensitivity.^12^ We therefore believe that CMB prevalence in TBI group cannot be attributed to age alone. Younger patients are more likely to undergo intensive conditioning regimens, including TBI, and may carry a significant burden of chronic HSCT related morbidity and late effects related to CMBs if future literature shows them to be clinically significant.

Our findings are of potential importance due to known association between CMBs and cognitive impairment,^15^ noting that multiple CMBs convey a stronger link.^7^ However, our study was underpowered to assess cognitive impairment. Furthermore, median latency since TBI‐conditioned HSCT in our study was 81 months while CMBs may predispose to cognitive impairment or dementia later in life. Studies with longer follow‐up and incorporating adequately powered cognitive testing would therefore be needed to investigate this. We would recommend that any studies with longer follow‐up also focus on cerebral vascular end‐points to further elucidate the clinical significance of our observations. Due to a small and heterogeneous sample, we were unable to assess the role of underlying disease, which may become a focus of future research.

This study provides the first evidence of increased CMB burden in TBI recipients. The doses used in TBI are much lower than those implicated in irradiation‐induced CMB formation thus far. Our data suggest that TBI may be particularly associated with development of multiple CMBs in young survivors, above and beyond what would be considered normal for their age. Small sample size is a marked limitation of our study and may in part explain failure to reach traditional thresholds of statistical significance. The main aim of further studies should be to validate present results, elucidate the impact of other peritransplant factors on CMB prevalence and explore clinically relevant end‐points such as cognitive impairment, dementia, stroke and intracerebral haemorrhage.

## AUTHOR CONTRIBUTIONS

**Asad Charania:** Conceptualization; investigation; methodology; project administration; data curation; resources. **Renuka Palanicawandar:** Methodology; writing – review and editing. **Ira Lacej:** Investigation; data curation. **Jiri Pavlu:** Writing – review and editing; methodology; formal analysis; project administration. **Jose Ros‐Soto:** Writing – review and editing. **Andrew Innes:** Writing – review and editing. **Jane Apperley:** Conceptualization; methodology; writing – review and editing. **Richard Szydlo:** Methodology; visualization; writing – review and editing; formal analysis; data curation; supervision. **Jure Hederih:** Conceptualization; investigation; writing – original draft; methodology; visualization; writing – review and editing; formal analysis; project administration; data curation. **Camelia Vladescu:** Conceptualization; methodology; resources; data curation; project administration; writing – review and editing. **Dragana Milojkovic:** Writing – review and editing. **Nichola Cooper:** Validation; conceptualization; investigation; funding acquisition; methodology; writing – review and editing; formal analysis; project administration; supervision; resources. **Adam Waldman:** Conceptualization; investigation; writing – review and editing; methodology; formal analysis; project administration; supervision; data curation; validation. **Eduardo Olavarria:** Conceptualization; methodology; writing – review and editing.

## FUNDING INFORMATION

The funder of this study was Imperial Biomedical Research Centre.

## CONFLICT OF INTEREST STATEMENT

A.C. has started employment with Roche after the completion of his involvement in this study. Other authors declare no relevant conflict of interest for this study.

## Supporting information


**Figure S1.** Cerebral microbleeds (white arrows) as seen on a transverse section of a SWI MRI image from a study participant with a history of total body irradiation‐conditioned haematopoietic stem cell transplant.
**Table S1.** Summary of Spearman rank correlation coefficients between selected variables and total CMB number.
**Table S2.** Summary of irradiation doses received by patients in the TBI group.
**Table S3.** Summary of acute^1^ and chronic^2^ GVHD prevalence, severity and prophylactic medication among the study participants.

## Data Availability

Please contact the corresponding author for deidentified data.
