# Supplementary material for: Total body irradiation is associated with increased burden of cerebral microbleeds in haematopoietic stem cell transplant recipients
Source: Br J Haematol. 2026 Aug 12;209(4):1923–7. doi: 10.1111/bjh.70747 (PMC13674133; doi:10.1111/bjh.70747)
Supplement: Supplementary file 1 [file BJH-209-1923-s001.docx]

**Supplementary Data**

Figure 1: Cerebral microbleeds (white arrows) as seen on a transverse section of a SWI MRI image from a study participant with a history of total body irradiation -conditioned haematopoietic stem cell transplantat.

|  | **r_s_** | **p** |
| --- | --- | --- |
| **BMI** | -0.25 | 0.37 |
| **aaHCT-CI** | 0.11 | 0.70 |
| **acute GVHD grade** | -0.13 | 0.65 |
| **Age at scan** | 0.57 | 0.03* |
| **Age at transplant** | 0.50 | 0.06 |
| **Latency since transplant** | 0.45 | 0.09 |
| **Nadir platelet count** | 0.02 | 0.93 |
| **Time to platelet engraftment** | 0.08 | 0.78 |

Table 1: Summary of Spearman rank correlation coefficients between selected variables and total CMB number. aaHCT-CI – age adjusted haemotopoietic cell transplant comorbidity index; GVHD – graft versus host disease; r_s_ – Spearman rank correlation coefficient; * - statistically significant result at p<0.05 threshold

|  |  | Number of patients (%) |
| --- | --- | --- |
| Total TBI dose | 12 Gy | 2 (22) |
|  | 13.2 Gy | 5 (56) |
|  | 14.4 Gy | 2 (22) |

Table 2: Summary of irradiation doses received by patients in the TBI group. All doses were fractionated; 12 Gy delivered as 220 cGy BD, 13.2 Gy as 200 cGy BD and 14.4 Gy as 180 cGy BD. TBI – total body irradiation

|  | **Chemotherapy + TBI** | **Chemotherapy only** |
| --- | --- | --- |
| **Total number of patients** | 9 | 6 |
| **Any GVHD** | 5 | 4 |
| **Acute GVHD** | | |
| Number of patients | 4 | 4 |
| Grade 1 | 1 | 1 |
| Grade 2 | 3 | 3 |
| Grade >2 | 0 | 0 |
| **Chronic GVHD** | | |
| Number of patients (score) | 1 (mild) | 1 (severe) |
| **GVHD prophylaxis** | | |
| Ciclosporin | 9 | 6 |
| Methotrexate | 9 | 2 |
| PT-Cyclophosphamide | 0 | 4 |
| MMF | 0 | 4 |

Table 3: Summary of acute[1] and chronic[2] GVHD prevalence, severity and prophylactic medication among the study participants. GVHD – graft versus host disease; PT – post-transplant; MMF – mycophenolate mofetil

**References**

[1]	D. Przepiorka *et al.*, “1994 Consensus Conference on Acute GVHD Grading,” *Bone Marrow Transplant*, vol. 15, no. 6, pp. 825–828, Jun. 1995.

[2]	M. H. Jagasia *et al.*, “National Institutes of Health Consensus Development Project on Criteria for Clinical Trials in Chronic Graft-versus-Host Disease: I. The 2014 Diagnosis and Staging Working Group report,” *Biol Blood Marrow Transplant*, vol. 21, no. 3, pp. 389-401.e1, Mar. 2015, doi: 10.1016/j.bbmt.2014.12.001.
